# Impacts of the COVID-19 economic slowdown on soybean crop yields in the United States

**DOI:** 10.1038/s41598-023-39531-6

**Published:** 2023-08-03

**Authors:** Julianna Christopoulos, Daniel Tong, Patrick C. Campbell, Siqi Ma

**Affiliations:** 1 National Oceanic and Atmospheric Administration, Climate Program Office, Silver Spring, MD 20910 USA; 2 Department of Atmospheric and Oceanic Sciences, University of California, Los Angeles, Los Angeles, CA 90095 USA; 3 Department of Atmospheric, Oceanic and Earth Sciences, George Mason University, Fairfax, VA 22030 USA; 4 Center for Spatial Information Science and Systems, George Mason University, Fairfax, VA 22030 USA; 5 National Oceanic and Atmospheric Administration Air Resources Laboratory, College Park, MD 20740 USA

**Keywords:** Atmospheric chemistry, Environmental impact

## Abstract

It is without question that the COVID-19 pandemic has taken its toll on the U.S. economy. Stay-at-home orders led to reduced vehicular traffic and widespread declines in anthropogenic emissions (e.g., nitrogen oxides (NO_x_)). This study is the first to explore the potential consequences of O_3_ changes resulting from the economic shutdown in the United States on soybean crop yields for 2020. The pandemic’s impact on surface O_3_ is quantified using the NOAA’s National Air Quality Forecasting Capability (NAQFC), which is based on the Community Multi-Scale Air Quality (CMAQ) model for May–July 2020. The “would-be”, 2020 level business-as-usual (BAU) emissions are compared to a simulation that uses representative COVID-19 (C19) emissions. For each emissions scenario, crop exposures are calculated using the AOT40 cumulative exposure index and then combined with county-level soybean production totals to determine regional yield losses. Exposure changes ranged between – 2 and 2 ppmVhr^−1^. It was further shown that increased exposures (0.5 to 1.10 ppmVhr^−1^) in the Southeast U.S. counteracted decreased exposures (0.8 to 0.5 ppmVhr^−1^) in the other soybean-producing regions. As a result, corresponding yield improvements counteracted yield losses around the Mississippi River Valley and allowed for minimal improvements in soybean production loss totaling $6.5 million over CONUS.

## Introduction

On January 30th, 2020, the World Health Organization (WHO) declared the outbreak of COVID-19 to be a Public Health Emergency of International Concern, posing a high risk to countries with vulnerable health systems^1^. The measures taken to contain the virus resulted in widespread changes in anthropogenic emissions. In early March 2020, state governments began issuing strict stay-at-home orders to contain the spread of the virus. As a result, widespread declines in anthropogenic emissions occurred and continued for the months that followed^2–4^. The most notable changes in pollutants occurred in the urban areas of the country, with nitrogen oxide (NO_x_) concentrations declining significantly as recorded from the collocation of both satellite- and ground-based observations^5,6^.

Locally, near-surface ozone (O_3_) is mainly formed through the photooxidation of precursor gases and volatile organic compounds (VOCs) in the presence of nitrogen oxides (NOx)^7^. O_3_ is not only detrimental to human health, resulting in diminished lung health function, but significantly hinders the growth of many plant species. As a result, O_3_ causes a wide variety of damage to agricultural crops including visible injury, reduction in photosynthesis, alterations to carbon allocation, and reduction in yield quantity and quality^8^. One study found that choosing crop varieties that are more ozone-resistant could improve global crop production in 2030 by 12% relative to the year 2000 level^9^. Currently, a variety of significant crop species are impacted by O_3_ exposures annually. As indicated by National Crop Loss Assessment Network (NCLAN) studies, dicot crop species (i.e., soybean, cotton, and peanut) are more sensitive to yield loss induced by O_3_ exposures compared to monocot crop species (i.e., sorghum, field corn, and winter wheat)^10^. Soybeans are a significant agricultural product in the U.S. The U.S. is currently the leading producer and second-leading exporter of soybeans. They constitute up to 90% of all oilseed production in the country and are among the most sensitive to O_3_ exposures (ERS, 2022). In 2005, exposure to ambient O_3_ was estimated to have reduced U.S. soybean production by 10% on a national average^11^. These factors make soybean an ideal crop for studying O_3_ impacts caused by COVID-19-related emissions changes. The impacts of COVID-19 related emission changes on soybean crop yields during the O_3_ photochemical season in the U.S. is currently unknown.

In this paper, we focus on the effects of the COVID-19 pandemic on air quality and agricultural production in the U.S. This study utilizes NOAA’s National Air Quality Forecasting Capability (NAQFC), based on the Community Multiscale Air Quality Model (CMAQ), to predict the changes in O_3_ concentration brought about by the pandemic^5^. We then use the NAQFC predicted O_3_ concentration changes and dose–response function relationships to quantify the pandemic-related changes on soybean crop yields for May–July (MJJ) 2020.

## Results

### Changes in ground-level O_3._

**Figure 1:**
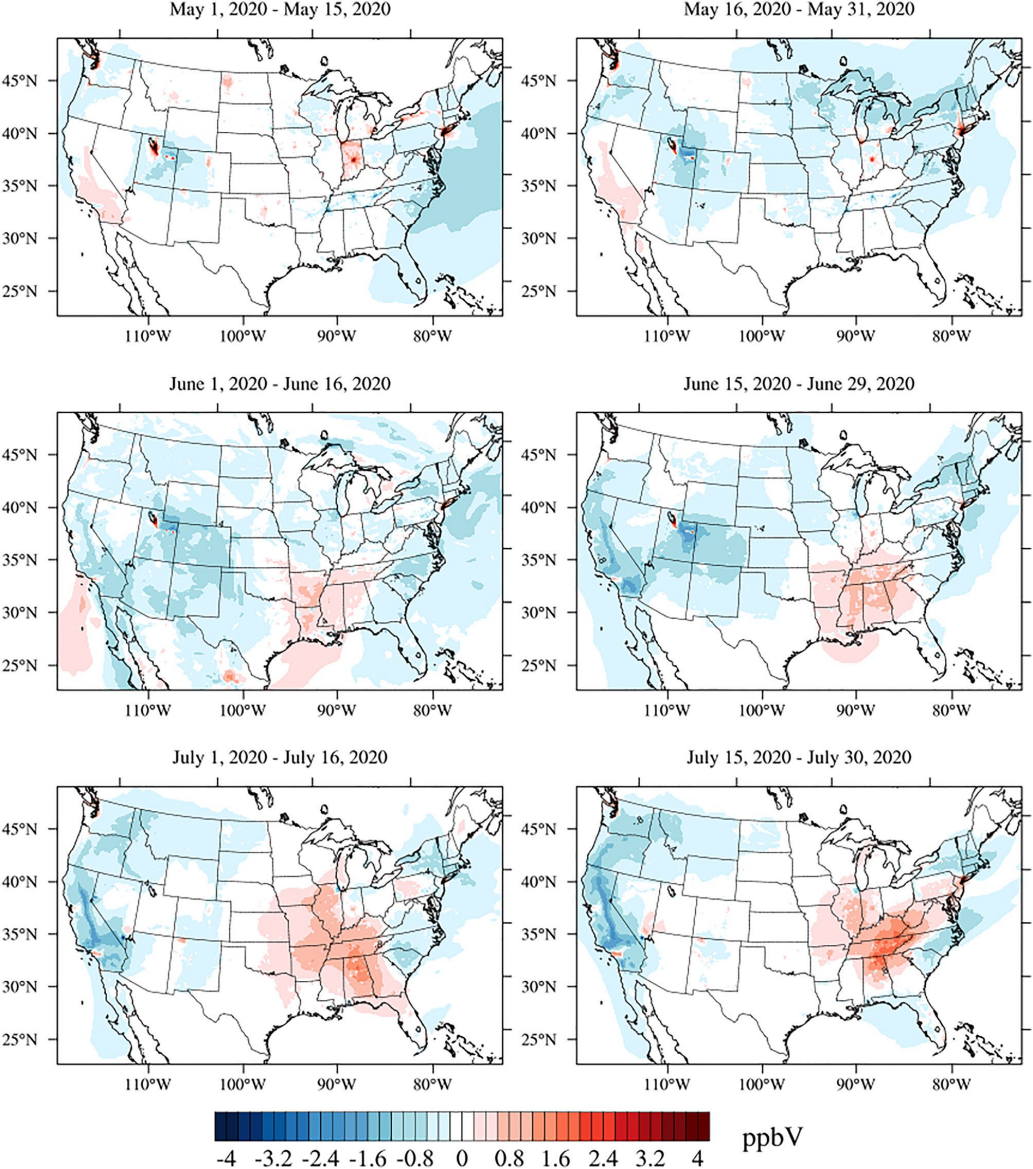
MJJ 2020 O_3_ concentration differences due to COVID-19 emissions changes (i.e., C19—BAU). Concentration changes varied by region with widespread decreases in the Midwestern U.S. of 1 ppbV (blue) and the Southeastern U.S. experiencing up to 3 ppbV increases (red). Created with NCL (NCAR Command Language) version 6.6.2. Available at: https://www.ncl.ucar.edu/^34^.

The differences in hourly O_3_ concentrations between a “business-as-usual” (BAU, i.e., the “would be” 2020 emissions without COVID-19 shutdowns) and actual COVID-19 (C19) scenarios are calculated and averaged for two-week periods for MJJ 2020 (Fig. 1; see Campbell et al.^5^ for scenario details). From MJJ, there are notable increases in O_3_, particularly in the Lower Midwest and Southeastern U.S. associated with NOx emissions increases among the rural NOx-limited regions (e.g., Midwest and Southeast). This relationship is not apparent for the major urban centers, which are VOC-limited during this time as suggested by the model^5^. A clear example is the area surrounding Indianapolis (VOC-limited), which shows increased O_3_ in May compared to the surrounding regions which show decreases in O_3_. Outside of the urban center, the region is more NO_x_-limiting allowing decreases in NO_x_ to drive a decrease in O_3_. Regarding the spatial variability of O_3_ changes_,_ the widespread increases in the Southeast regions were demonstrated to be in qualitative agreement with the U.S. EPA AirNow network observations (https://www.airnow.gov/) and simulated increases by NASA’s GEOS Composition Forecasting (GEOS-CF) system (see Campbell et al.^5^ for spatial variability). Increases can be attributed to rebounding emissions trends from MJJ when states began to lift restrictions^12^. In addition, shifts to cleaner vehicle fleets under the BAU scenario may have contributed to fewer emissions compared to C19’s shift to heavy-duty truck traffic during morning rush^5^. In other words, trucks played a larger role in the C19 scenario than in the BAU scenario. It is suspected that heavy duty trucks resulted in increased emissions due to increases in online consumption which continued from the later stages of the pandemic into the photochemical O_3_ season. Furthermore, under the BAU scenario, there was an expected 3–5% decrease in emissions due to the overturning of the vehicle fleet but COVID-19 interrupted this. It is important to note the uncertainty present due to background NO_2_ and natural variability which could play a role here as well (see “Discussion”).The regions along the Mississippi River Valley (e.g., Lower Midwest and Southeast), which were characterized by O_3_ increases, are of interest for this study since they occur throughout much of the soybean-producing region.

### Changes in crop exposures

**Figure 2:**
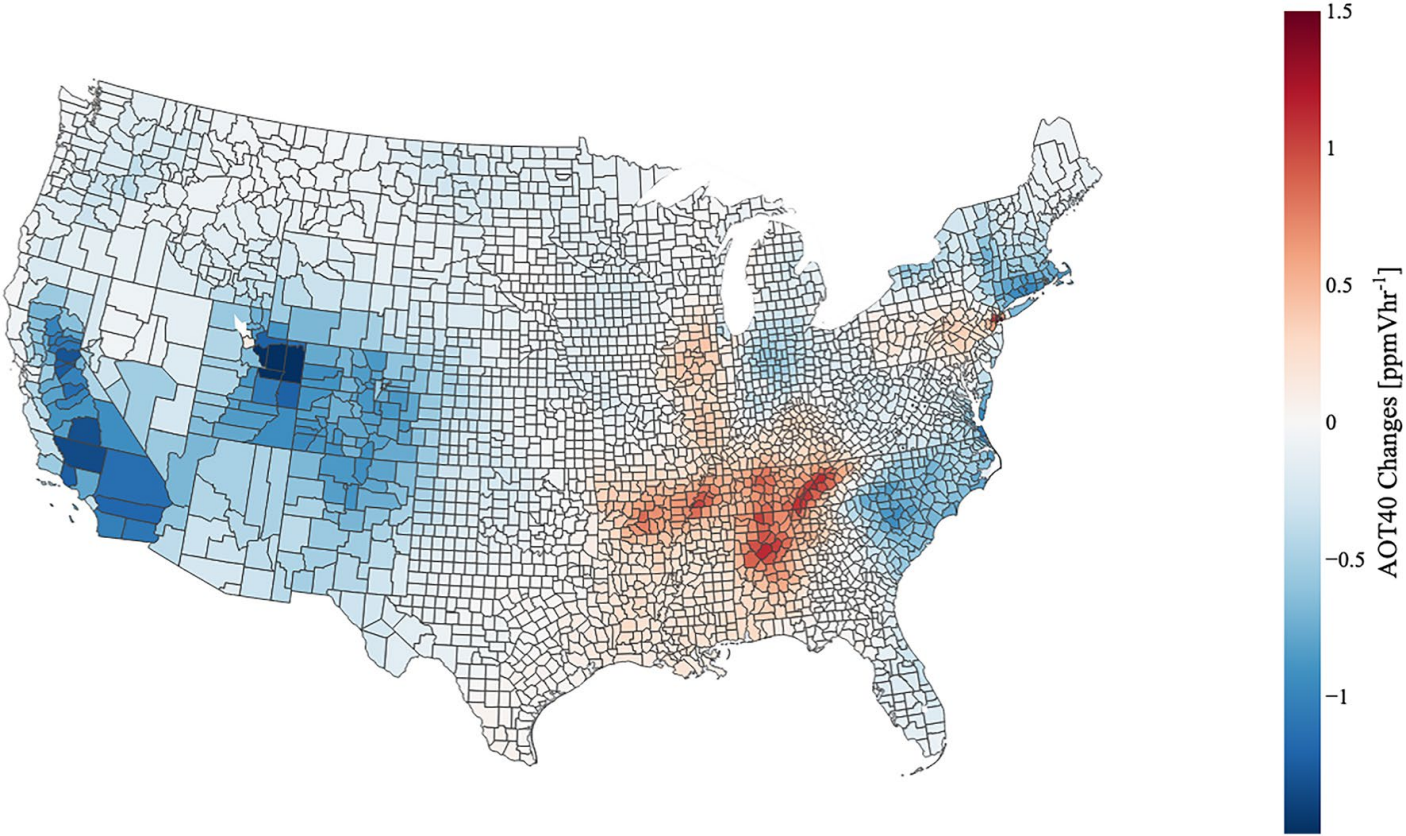
Differences in AOT40 indices between BAU and C19 (i.e., C19-BAU) scenarios by county. Regions of increased O_3_ exposures are depicted in the red counties while regions of decreased exposures are depicted in the blue counties. Created with Plotly version 5.14.1. Available at: https://plotly.com/python/^35^.

In both the BAU and C19 cases, highest exposures (based on the AOT40 metric; see “Methods” section) were concentrated in the Southwest regions of the U.S. with maximums occurring over southern California. Exposure differences between BAU and C19 are summarized by region in Table 1. The change in exposures induced by C19 are depicted in Fig. 2. The Western region experienced the maximum changes due to the emission changes with exposures decreasing by as much as 1.5 ppmVhr^−1^ in Central/Southern California and > 1.5 ppmVhr^−1^ in Utah. However, these regions do not contain soybean crops so they can be disregarded. The focus is on exposure changes occurring in the Midwest and Southeast regions which exhibit variable regional characteristics. In the Southeast, exposures both increased and decreased depending on the region/state. For example, in South Carolina and North Carolina exposures decreased (blue areas) by approximately 0.8 ppmVhr^−1^. Meanwhile, in the areas around Arkansas, Tennessee, and Kentucky, exposures increased (red areas) between 0.5 and 1.25 ppmVhr^−1^. The Midwest exhibited mainly decreased exposures (~ 0.75 ppmVhr^−1^ for Ohio, Indiana, Michigan and ~ 0.5 ppmVhr^−1^ for South Dakota and North Dakota). It is important to note, under the C19 emissions changes, the soybean-producing regions of the United States experienced nearly equivalent increases and decreases in O_3_ exposures (ranging between − 0.8 and 1.25 ppmVhr^−1^) that were regionally dependent (see Fig. 2) and nearly characteristic of the O_3_ changes determined for MJJ (see Fig. 1).

**Table 1:** Summary of regional O_3_ exposures (AOT40 in ppmVhr^−1^) and soybean yield losses (in bushels of soybean) due to C19 emissions changes.

	AOT40 (ppmVhr^−1^)	Yield loss changes (Bu)
Northeast	− 1.00 to 1.80	− 13.3 to 7.5 K
Midwest	− 0.60 to 0.45	− 40.5 to 82 K
Southeast	− 0.95 to 1.10	− 23 to 110 K
Southwest	− 0.70 to − 0.10	–
West	− 1.75 to 0.020	–

### Changes in yield loss

**Figure 3:**
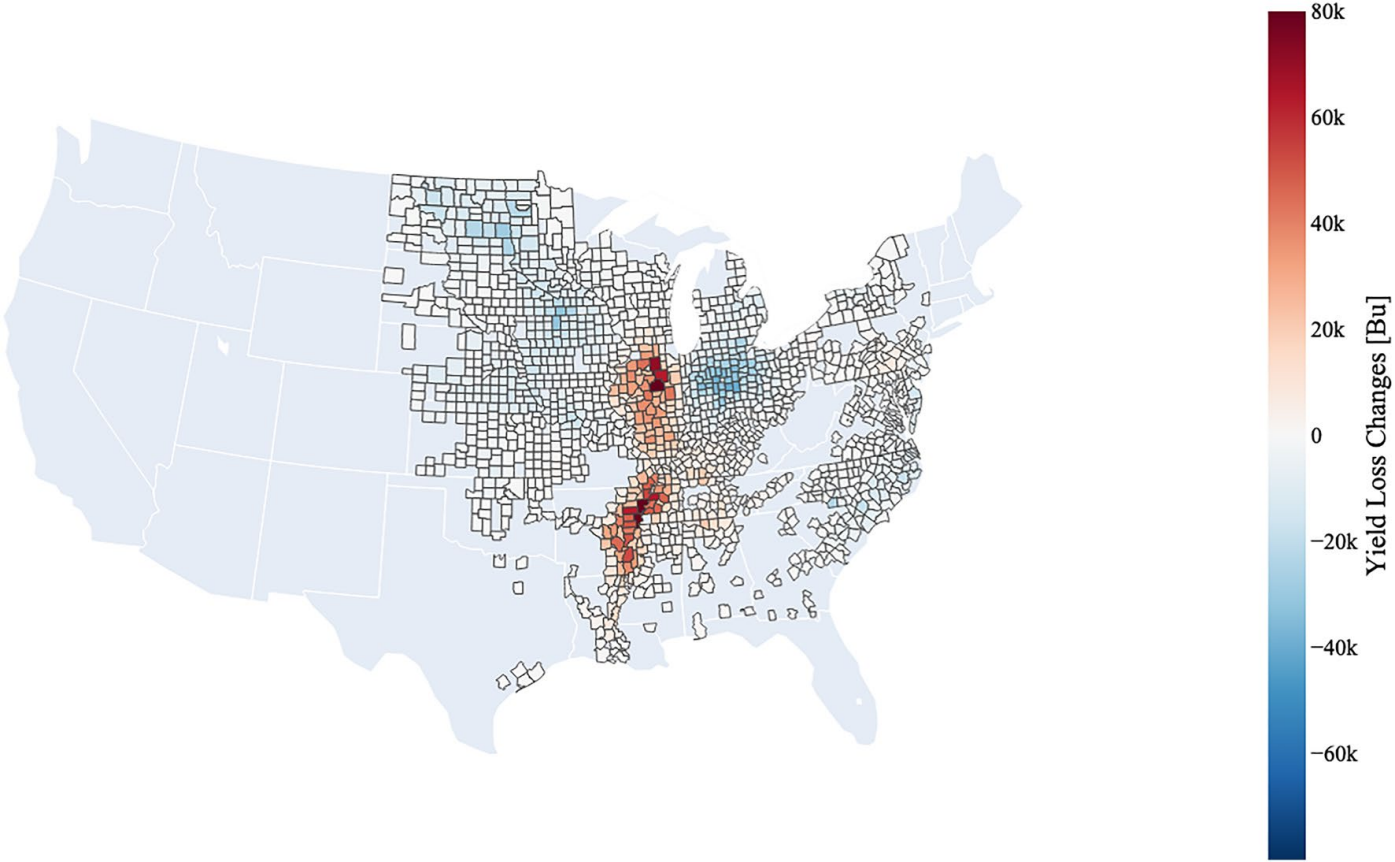
Differences in yield losses between BAU and C19 (i.e., C19-BAU) scenarios by county. Yield improvements (blue counties) are depicted in widespread areas in the Midwest and Southeast. Increased yield loss (red counties) under C19 is present along the Mississippi River Valley. Created with Plotly version 5.14.1. Available at: https://plotly.com/python/^35^.

Differences in C19 and BAU soybean yield losses are presented in Table 1 by region. Yield loss changes are nearly reflective of the exposure changes (see Figs. 2 and 3). Under C19, yield losses were significantly heightened (20 K Bu–110 K Bu) in counties along the Mississippi River, with individual counties in Arkansas and Illinois experiencing up to 80 K Bu and 110 K Bu in yield losses, respectively. It is important to note maximum yield losses occurred in counties with the highest production totals. Throughout the rest of the Midwest, yield improvements (blue areas) of 10 K Bu–40 K Bu are evident which offset the increases. The total U.S. soybean production reduction (%) in Table 2. represents the fraction of total yield loss to the total annual production. As a result, overall soybean production under the BAU and C19 scenarios was reduced by approximately 5.86% and 5.84% for MJJ 2020, respectively, an approximate 4% improvement from production losses since 2005^11^. These results are consistent with historical analyses, where production loss from O_3_ in the U.S. for 1980–2011 is estimated to have ranged between 4 and 6% on average^13^. In addition, they are consistent with Seltzer et al.^14^ (AOT40 RYL from soybean equivalent to 4.8% in 2015), Lobell et al.^15^ (5% average total soybean yield losses over last two decades), Da et al.^16^ (4.5% soybean annual production reduction for 1980–2015) and Liu and Desai^17^ (4.8% historic soybean RYL for 1980–2019).

**Table 2:** Summary of CONUS soybean losses.

	BAU SCENARIO	C19 SCENARIO
Total yield loss (Bu)	236,998,365	236,277,359
Average yield loss (Bu)	161,114	161,114
U.S. soybean production loss (%)	5.86%	5.84%
Difference in reduction (%)	0.020%	

In this work, it is important to note meteorological effects are not separated from Campbell et al.^5^ and as a result, meteorology may be a strong controlling factor over COVID-19 emissions changes on O_3_ concentration changes (and thus exposures) between 2019 and 2020. Isolating the meteorological vs. emissions impact on the COVID-19 related O_3_ changes is beyond the scope of this study, however state-level natural variability factors indicate less natural variability for the months of March–June 2020 with a larger contribution from natural variability for the later summer months (e.g., July–September). Larger contributions in natural variability indicate there are strong meteorological drivers of the NO_2_ precursor changes. This indicates O_3_ concentrations may be less impacted by the COVID-19 emissions changes for this period which in part agrees with Goldberg et al.^18^.

There are minimal differences present between the BAU and C19 yield loss, given nonlinearity in the C19 O_3_ concentration and exposure changes throughout the soybean-producing region for MJJ 2020. The regional decreases and increases in O_3_ exposures and yield losses work to offset one other. As a result, over CONUS, yield loss was slightly improved, by 0.02%, under the C19 scenario. The resulting economic effects of the changes in yield losses are summarized in Table 3, where soybean yield loss amounts were multiplied by the price in U.S. Dollars (USD) of U.S. soybeans per bushel. Production loss amounted to approximately $2.1 billion overall for the BAU and C19 emissions scenarios for 2020, also coinciding with the findings of Mcgrath et al.^13^ (annual soybean losses of $2 billion for the 31-year period) and Da et al.^16^ (average annual revenue losses of $1.2 billion for 1980–2015). A closer examination reveals a slightly improved production loss, amounting to $6.5 million, under C19 compared to the BAU emissions projections for 2020.

**Table 3:** Summary of CONUS production gains and losses due to O_3_ exposures.

U.S. soybean price per Bushel (as of August 21st, 2020)	$9.0075
BAU production loss (USD)	$2,134,762,777.00
C19 production loss (USD)	$2,128,268,215.00
Production gain attributed to COVID-19 emissions (USD)	$6,494,461.00

## Conclusion

This study quantifies the impacts of changes in ground-level O_3_ due to the COVID-19 (C19) pandemic on soybean crop yields for MJJ 2020. Compared to the “business-as-usual” (BAU) scenario, there were notable increases in O_3_ exposures which occurred in the Mississippi River Valley and southeast U.S., which can be attributed to increased NOx concentrations throughout the southern U.S. in July. The soybean-producing regions of CONUS saw regionally dependent changes in O_3_ exposures that were reflective of concentration changes shown for MJJ 2020. The Southeast and Midwest regions saw both increases and decreases in exposures (AOT40) that were equivalent in magnitude. Yield losses are reflective of these exposure changes with select counties in Arkansas and Illinois seeing increased yield losses up to 110 K Bu. Over CONUS, it is shown yield improvements counteracted by yield losses in the Mississippi River Valley regions allowed some improvement in production losses ($6.5 million USD compared to 2019) to have occurred as a result of the O_3_ concentration changes under C19. Overall, it was shown that 37% and 63% of the soybean-producing counties experienced yield loss increases and improvements, respectively. Yield improvements due to emission changes over CONUS represent 0.02% of the total 2020 U.S. soybean production which amounted $46 billion (https://www.nass.usda.gov/). Overall, while Campbell et al.^5^ highlighted the nonlinearity of O_3_ concentration changes due to the pandemic’s economic slowdown, here, we further show that these nonlinear changes result in regionally dependent O_3_ exposure changes (e.g., increases in the Southeast U.S. and widespread decreases elsewhere) throughout the soybean growing season.

Such results draw attention to the shift back to normalcy following the initial onset of the COVID-19 pandemic. If shutdown orders had not been relaxed at the same time as shifts to cleaner vehicle fleets under the BAU scenario during the summer months throughout the soybean-producing region, reduced vehicular traffic emissions would have contributed to decreased O_3_ exposures and improved yields. Throughout May, and the months that followed in our study, shutdowns were reduced throughout much of the U.S. resulting in the slightly changed chemistry we see for the summer months, despite reductions to overall vehicular traffic compared to 2019.

It will be necessary to further study the impact of economic-related emissions changes on crop yields, perhaps on longer time scales and to distinguish the effects of regional meteorology on yield losses in the future.

## Discussion

### Limitations of the modeling platform

There are also some challenges and limitations in the National Air Quality Forecast Capability (NAQFC) O_3_ simulations that drive the impacts on crop exposures and yields in this paper. Campbell et al.^5^ showed that there are widespread O_3_ decreases in the U.S. rural regions (typically NOx limited; with lower COVID-19 NOx emissions), and instances of relatively localized O_3_ increases in and around urban regions (typically VOC-limited; but also, with lower COVID-19 NO_x_ emissions). These O_3_ changes strongly rely on the BAU and C19 emission projection scenarios derived from ground-(U.S. EPA Air Quality System network) and satellite-based (Aura Ozone Monitoring Instrument) observations, but in rural regions there is relatively sparse coverage and a low sensitivity of OMI to capture small surface O_3_ changes. This can lead to additional uncertainties in the derived NOx emissions adjustment factors for C19 and the resulting O_3_ concentration changes in rural regions. An example of this is the South/Southeast U.S. where there are widespread larger C19 emissions compared to the BAU case after June, resulting in increased O_3_ in the widespread NO_x_-limited regions. It is difficult to fully assess if such widespread O_3_ increases occurred with relatively minimal point observations to compare with, but comparisons with the U.S. EPA AirNow observations in Campbell et al.^5^ in part support this change. Qu et al.^6^ approximate the impacts of natural/background effects when using NO_2_ observations to infer NO_x_ emissions changes during the pandemic. They found that the satellites show much weaker NO_2_ responses in March–June and no decrease in July–August, consistent with a large background contribution to the NO_2_ column in the U.S. This partly confounds the use of OMI in deriving the C19 NO_x_ emissions changes and adds some inherent uncertainty to our work here. However, a detailed investigation into the natural variability is beyond the scope of Campbell et al.^5^ or in this paper. Hence, the reader is further referred to Goldberg et al.^18^ and Qu et al.^6^ for detailed analyses of satellite observations and the natural variability impacts on NO_2_ concentration changes during the COVID-19 lockdown.

## Methods

### Air quality model configuration

The NWS/NOAA National Air Quality Forecasting Capability (NAQFC) used in this work is a well-documented and evaluated air quality modeling system^19–23^, and the experimental version used here is based on the offline-coupled North American Mesoscale Model Forecast System on the B-Grid (NMMB)^24,25^, which provides the driving weather data to the CMAQ model, version 5.0.2^26^. The domain of the NAQFC covers the continental U.S. (CONUS) at a horizontal grid resolution of 12 × 12 km with 35 vertical levels. CMAQ simulates the formation, transport, and fate of a suite of atmospheric composition parameters. The NAQFC has provided real time air quality forecast guidance for over the past decade for different EPA-defined criteria pollutants including O_3_ at a horizontal resolution of 12 × 12 km centered over CONUS. The full NAQFC model configurations and inputs are described in Campbell et al.^5^. BASE (NEI2014v2) O_3_ simulations compared against the U.S. EPA AirNow observations for April-September 2020 were indicative of acceptable (i.e., consistently fall within the criteria ranges for O_3_ established by Emery et al.^29^) model performance with little exceptions. For the detailed comparison, see Campbell et al.^5^.

### Emission changes caused by COVID-19

The emission changes caused by COVID-19 are derived from the difference from two scenarios: a “business-as-usual” (BAU) case and a COVID-19 (C19) case. In the BAU case, the emission data from the NEI 2014 version 2 (NEI2014v2) (i.e., the baseline emissions) are projected into the “would-be” 2020 level by using the mean rate of NO_2_ trends observed from satellite and ground sensors for the period of 2014–2019, the year before the pandemic. In the C19 case, the observed NO_2_ trends from 2014 to 2020, which are based on the vertical column density of NO_2_ from the Ozone Monitoring Instrument (OMI) aboard the Aura satellite, and the U.S. EPA Air Quality System ground network NO_2_ observations, are used to represent the actual emission level under the pandemic conditions. For both cases, the NO_2_ trend data are derived using the approach developed by Tong et al.^27,28^. Detailed data processing and quality control procedures are provided in Tong et al.^27^. The emission data after adjustment are then used to drive the chemical transport model component of NAQFC, i.e., CMAQ, to calculate the near-surface O_3_ levels under each scenario. The difference between the predicted O_3_ concentrations in between BAU and C19 is attributed to the impact of the pandemic. Evaluation of the NAQFC surface O_3_ concentrations using the NEI2014v2 (i.e., baseline emissions), BAU, and C19 scenarios demonstrated that the monthly MJJ 2020 model performance for surface O_3_ was within statistical benchmark criteria defined by Emery et al.^29^. Additionally, the BAU and C19 O_3_ simulations displayed increased in correlation, R, Index of Agreement (IOA), and decreased Normalized Mean Error (NME) compared to the BASE case. (see Campbell et al.^5^ for the full NAQFC statistical evaluation) Further details on the BAU and C19 emissions used in this work, as well as the state-level emission adjustment factors for scenarios of C19 and BAU in MJJ 2020 are found in Campbell et al.^5^.

### Examination of soybean crop exposures

To examine what effect the changes in the modeled NAQFC O_3_ concentrations may have had on soybean crop yields, it is necessary to evaluate the degree to which crops are being exposed to O_3_. Assessments of crop loss from O_3_ exposures in the U.S. are based on dose–response function relationships. The soybean exposures are calculated using exposure indices that are related to those dose–response relationships from which yield losses are derived^11^. For this study we utilize an index to quantify the accumulated O_3_ exposure over a threshold of 40 ppb^8^, AOT40, which is defined as:

1 $$AOT40\;\left( {ppmVhr^{ - 1} } \right) = \mathop \sum \limits_{i = 1}^{n} [C_{{O_{3} }} - 0.04] \;for\; CO_{3} \ge 0.04 \;ppm$$

The AOT40 index represents the sum of positive differences between the hourly mean O_3_ concentration (${C}_{{O}_{3}}$) and a threshold of 0.04 ppm, multiplied by the 1-h averaging period (n), in a fixed growing season^30^. The cutoff at 0.04 ppm or 40 ppb is based largely on the anthropogenic component of the ozone exposure and does not imply a threshold for biological effects^31^. In this study, we calculate AOT40 for 24-h periods in three consecutive months of the growing season under the BAU and C19 scenarios. AOT40 is calculated for MJJ. It is important to note, O_3_ damage accumulates over the growing season^32^. The earlier months of the growing season are used to examine the potential effects produced from the shutdown-related emission changes. Since crop production data in the U.S. is based on the county-level, the AOT40 indices are converted to a county-level average. O_3_ concentrations of all related grid cells are averaged into a single county and weighted by area for consistency following the approach of Tong et al.^11^.

### Calculation of crop yield loss

The dose–response function for AOT40 is based on a linear relationship for soybeans from Dingenen et al.^33^. The relative yield loss (RYL), is calculated using this dose–response function and is defined as:

2 $$RYL \left( {ppmVhr^{ - 1} } \right) = a \times AOT40$$

where the constant a = 0.0113 is determined from Dingenen et al.^33^ as a simple relationship between AOT40 and soybean crop yields. Soybean production amounts for 2020 (bushels of soybean per county) are obtained from the U.S. Department of Agriculture National Agricultural Statistics Service (https://www.nass.usda.gov/Data_and_Statistics/) (see Table 4, for total production). A small amount of production data from combined counties is excluded since data from those individual counties could not be determined given individual farmer’s privacy. The RYL values are then combined with the 2020 soybean production (approximately 4 billion bushels with the subtracted counties) to generate the actual yield loss in Bu.

**Table 4:** Summary of 2020 CONUS soybean production data.

	2020
Total production (USD)	$45,732,122,000
Total production (Bu)	4,216,302,000

## Data Availability

All crop yield data used in this study are openly available from the United States Department of Agriculture’s National Agriculture Statistics Service at https://www.nass.usda.gov/index.php. Dataset information for modeling inputs and observations are included in Campbell et al.^5^.
